# Active Compression During External Cardioversion of Atrial Fibrillation: A Meta‐Analysis of Randomized Controlled Trials

**DOI:** 10.1111/anec.70074

**Published:** 2025-04-01

**Authors:** Hosam I. Taha, Abubakar Nazir, Ahmed A. Ibrahim, Mohamed S. Elgendy, Abdalhakim Shubietah, Hazem Reyad Mansour, Sherif Sary, Moataz Maged, Mustafa Turkmani, Mohamed Abuelazm

**Affiliations:** ^1^ Faculty of Medicine Tanta University Tanta Egypt; ^2^ Faculty of Medicine King Edward Medical University Lahore Pakistan; ^3^ Faculty of Medicine Menoufia University Menoufia Egypt; ^4^ Department of Internal Medicine Advocate Illinois Masonic Medical Center Chicago Illinois USA; ^5^ Faculty of Medicine Michigan State University East Lansing Michigan USA; ^6^ Department of Internal Medicine McLaren Health Care Oakland Michigan USA

**Keywords:** arrhythmia, atrial fibrillation, chest pressure, defibrillation, electric countershock, resuscitation

## Abstract

**Objectives:**

Direct current cardioversion (DCCV) is commonly used for atrial fibrillation, but there is uncertainty about whether active chest compression improves its effectiveness. This meta‐analysis evaluates the impact of active compression on cardioversion outcomes.

**Methods:**

A systematic review and meta‐analysis synthesizing evidence from randomized controlled trials (RCTs) retrieved from PubMed, Scopus, WOS, Embase, and Cochrane Library till September 2024. Statistical analysis was performed using R software (version 4.3.1), applying risk ratios (RR) for dichotomous outcomes and mean differences (MD) for continuous outcomes, with 95% confidence intervals (CI). PROSPERO ID: CRD42024595499.

**Results:**

Four RCTs with 737 patients were included. When compared to the no‐compression approach, active compression during DCCV was not associated with any significant difference in cardioversion success (RR: 1.10; 95% CI [0.96, 1.25], *p* = 0.16), first shock success (RR: 1.62; 95% CI [0.94, 2.81], *p* = 0.08), number of shocks (MD: ‐0.32; 95% CI [−1.01, 0.36], *p* = 0.36), or crossover success (MD: 0.76; 95% CI [0.33, 1.77], *p* = 0.52). However, active compression was associated with a reduced successful shock energy (MD: ‐23.97 J; 95% CI [−26.84, −21.10], *p* < 0.01).

**Conclusion:**

Active compression during DCCV does not significantly improve cardioversion success but may reduce the energy required for successful cardioversion, suggesting potential safety benefits. However, further studies are needed to determine its clinical relevance.

## Graphical abstract

This meta‐analysis of four RCTs (*n* = 737) evaluates the impact of active compression during DCCV for atrial fibrillation. Active compression does not significantly improve cardioversion success but reduces the energy required for successful defibrillation (MD: −23.97 J; 95% CI [−26.84, −21.10], *p* < 0.01), suggesting potential safety benefits that warrant further investigation.

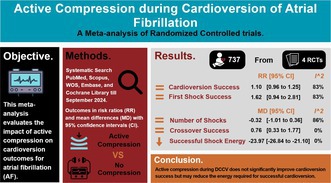

## 1 Introduction

Atrial fibrillation (AF), the most common cardiac arrhythmia, affects an estimated 37 million people globally (Lippi et al. 2021; Amin, Ghaly, et al. 2024). It is projected that 6 to 12 million individuals in the United States will be affected by this condition by the year 2050, while an estimated 17.9 million individuals in Europe are anticipated to be affected by 2060 (Lippi et al. 2021; Amin, Nazir, et al. 2024). By 2050, projections indicate a 60% increase in cases, highlighting its growing impact on aging populations (Lippi et al. 2021; Elliott et al. 2023). The prevalence of AF rises from 1.5%–2% in the general population to nearly 10% among those over 80 years (Klein and Trappe 2015). This arrhythmia is strongly associated with cardiovascular complications, including a five‐fold increase in stroke risk, heart failure, and increased hospitalization (Elliott et al. 2023; Klein and Trappe 2015; Ali et al. 2006).

Direct current cardioversion (DCCV), an essential strategy to restore sinus rhythm, achieves success in 90% of patients, but AF recurrence rates hover around 50% within a year (Ferreira et al. 2024). Applying pressure on defibrillator pads reduces transthoracic impedance, enhancing energy transfer and potentially improving cardioversion outcomes by lowering impedance (Ferreira et al. 2024). However, active compression can cause discomfort and complications like skin burns (Ferreira et al. 2024).

Emerging evidence supports the efficacy of non‐compression techniques, particularly with modern biphasic waveform defibrillators, which have shown superior performance in AF conversion without the need for compression (Link et al. 2010). These developments have spurred interest in less invasive, patient‐friendly approaches to cardioversion (Link et al. 2010).

Our systematic review and meta‐analysis will compare the efficacy of active compression versus the no‐compression approach during AF cardioversion. We will examine key outcomes such as cardioversion success, number of shocks, and energy required for cardioversion success. This study will provide evidence‐based recommendations for refining cardioversion protocols to improve patient care, focusing on minimizing recurrence, discomfort, and complications while maximizing procedural success.

## 2 Methodology

### 2.1 Protocol Registration

This systematic review and meta‐analysis was conducted according to the PRISMA guidelines (Page et al. 2020). In addition, we adhered to the Cochrane Handbook for Systematic Reviews of Interventions (Higgins et al. 2024). We registered the protocol in PROSPERO under the ID: CRD42024595499.

### 2.2 Data Sources and Search Strategy

Two authors (H.I.T. and M.S.E.) independently conducted a comprehensive search on September 24th, 2024, across PubMed, Cochrane Library, Scopus, WOS, and Embase without restrictions on data or language. We included both free‐text keywords and MeSH terms: (“pressure” OR “compression”) AND (“cardioversion” OR “defibrillation”) AND (“atrial fibrillation”). (H.I.T.) manually checked references to ensure no relevant articles were missed. The search details can be found in Table A.1 in Appendix S1.

### 2.3 Eligibility Criteria

Studies that met the following PICOS criteria were included: *population (P)*: adult patients ≥ 18 years with persistent AF; *intervention (I)*: active compression during DCCV; *control (C)*: no compression during DCCV; *outcomes (O)*: cardioversion success, first shock success, second shock success, third shock success, fourth shock success, number of shocks, crossover success, total energy delivered, and successful shock energy; *study design (S)*: randomized controlled trials (RCTs).

We excluded animal studies, in vitro studies, case–control studies, cohort studies, non‐randomized trials, feasibility studies, reviews, books, theses, case reports, case series, unpublished work, protocols without results, studies with incomplete or duplicate data, and conference abstracts. Studies in which cardioversion was done for indications other than AF were excluded. Studies in which the crossover was done between groups other than the active and no‐compression groups were also excluded.

### 2.4 Study Selection

After duplicates were removed via EndNote (version 20.4.1), all remaining records underwent title and abstract screening independently by three reviewers (H.R.M., S.S., and M.M.) using Rayyan. Reference screening was done by a fourth author (H.I.T.) to ensure no relevant studies were missed. Studies meeting inclusion criteria underwent full‐text review, and (H.I.T.) resolved any discrepancies.

### 2.5 Data Extraction

Eligible full texts underwent pilot data extraction to format the data extraction sheet on Excel (Microsoft, USA). (M.S.E., H.R.M., S.S., and M.M.) independently extracted data into three sections: (1) a summary of included studies, including the name of the first author, study design, country, number of centers, total sample size, electrode position, number of shocks delivered before crossover, energy sequence of shocks, primary outcome, and inclusion criteria; (2) baseline participant characteristics: (demographics, AF duration, and past medical history); and (3) outcomes data on efficacy, as previously mentioned. Successful shock energy, in our analysis, refers to the minimum amount of electrical energy required to successfully convert the heart's rhythm from AF to normal sinus rhythm.

### 2.6 Risk of Bias and Certainty of Evidence

The Cochrane Risk of Bias 2 tool (Sterne et al. 2019) was used to assess the included RCTs' quality. It measures five key domains, including biases in randomization, deviations from the intended intervention, missing outcome data, outcome measurement deviations, and selective reporting of results (Sterne et al. 2019). The studies were then categorized as having “low risk”, “some concerns”, or “high risk”. Four reviewers (H.I.T., M.S.E., S.S., and H.R.M.) conducted this assessment, and discrepancies were resolved via discussion with an additional reviewer (M.A.). Although publication bias was considered, it was unreliable because fewer than 10 studies were included (Sterne et al. 2019).

The certainty of the evidence was assessed by (M.S.E.) using the Grading of Recommendations, Assessment, Development, and Evaluation (GRADE) system (Guyatt et al. 2008). This method considers five factors: risk of bias, inconsistency, indirectness, imprecision, and other relevant factors. After evaluation, we assigned an overall quality rating of the evidence to be high, moderate, low, or very low certainty (Guyatt et al. 2008). Finally, the grading was reviewed for accuracy by (M.A.).

### 2.7 Statistical Analysis

The statistical analysis was conducted by (A.A.I.) using R software version 4.3.1. For dichotomous outcomes, the risk ratio (RR) was used. In contrast, for continuous outcomes, the mean difference (MD) was used, both with 95% confidence intervals (CI) using the random‐effects model when there was a significant heterogeneity (*I* ^2^ > 50%) and the common‐effect model when heterogeneity was not significant (*I* ^2^ < 50%). Heterogeneity was assessed using chi‐square and I^2^ statistics. The chi‐square test shows the presence of heterogeneity, and the I^2^ statistic shows the degree of heterogeneity. We used an alpha level below 0.1 for the Chi‐square test to interpret significant heterogeneity. We conducted a Leave‐one‐out sensitivity analysis to assess our findings' robustness for outcomes with significant heterogeneity (*I* ^2^ > 50%). This approach involves iteratively excluding one study at a time and recalculating the pooled effect size. Data presented as median with interquartile range underwent post hoc analysis, converting it to mean with standard deviation. Finally, we conducted a meta‐regression analysis based on the study‐level covariate (body mass index, BMI).

## 3 Results

### 3.1 Search Results and Study Selection

**FIGURE 1:**
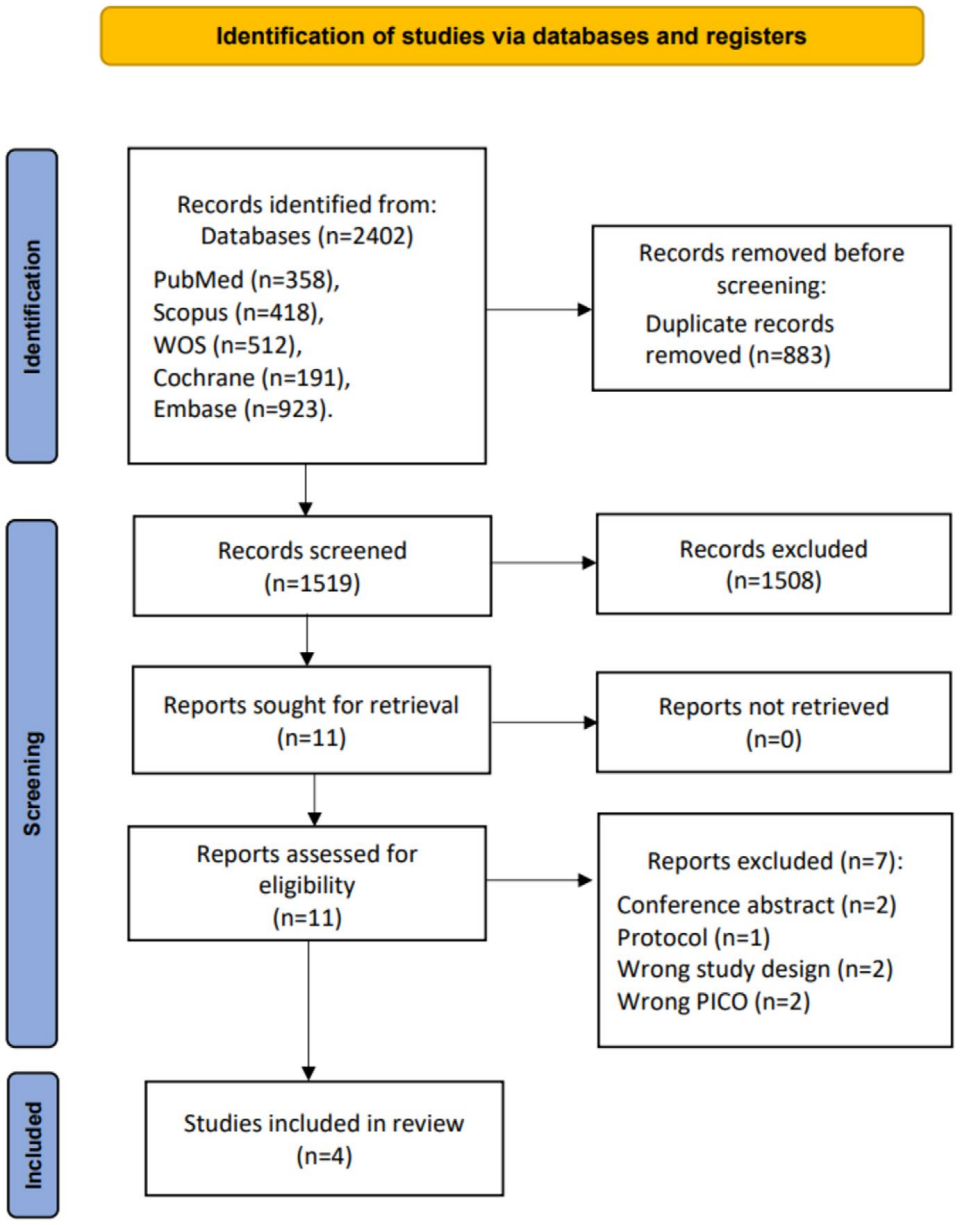
PRISMA flow chart of the screening process.

We identified 2402 studies after searching databases. Duplicate removal via EndNote excluded 883 duplicates, leaving 1519 studies for title and abstract screening. Of these, 1508 did not meet our inclusion criteria and were excluded. We evaluated the full texts of the remaining 11 studies in detail. Ultimately, seven studies (Dodd et al. 2004; Ramirez et al. 2016; Lakananurak et al. 2022; Ferreira et al. 2021; Forgione et al. 2000; Kerber et al. 1981; Cohen et al. 1997) were removed, as illustrated in Table A.2 in Appendix S1, leaving four studies (Ferreira et al. 2024; Squara et al. 2021; Kirchhof et al. 2005; Voskoboinik et al. 2018) that met all the inclusion criteria. A PRISMA flow diagram demonstrates the study selection process (Figure 1).

### 3.2 Characteristics of Included Studies

Four RCTs with a total of 737 patients were included. Of these, 374 were assigned to the active compression group, and 363 underwent the no‐compression approach. Three trials (Ferreira et al. 2024; Squara et al. 2021; Voskoboinik et al. 2018) were multicenter, whereas one RCT was a single‐center trial (Kirchhof et al. 2005). These RCTs were conducted in Australia, France, and Germany. Blinding status differed between the studies. Additionally, the studies applied different energy sequences for DCCV. Detailed summaries of the included RCTs and baseline participant characteristics are presented in Tables 1 and 2.

**TABLE 1:** Summary characteristics of the included RCTs.

Study ID	Study design	Country	*N*. of centers	Sample size, *N*.	Electrodes position and type of shock	*N*. of shocks delivered before crossover	Energy sequence of shocks, Joules	Primary outcome	Inclusion criteria
Ferreira et al. (2024)	Double‐blinded, RCT	Australia	3	311	AP biphasic	3	150, 200, 360	Total energy delivered in Joules	Age ≥ 18, confirmed AF on ECG, no evidence of LA thrombus, ≥ 3 weeks of anti‐coagulation and ability to provide informed consent
Squara et al. (2021)	Unblinded, RCT	France	2	100	AP biphasic	4	50, 100, 150, 200	Defibrillation threshold	Age ≥ 18 with persistent AF ≥ 7 days
Voskoboinik et al. (2018)	Unblinded, RCT	Australia	4	125	AA or AP biphasic	2	100, 200	Cardioversion success^a^	Obesity (BMI ≥ 30) and persistent AF undergoing clinically indicated external cardioversion
Kirchhof et al. (2005)	Unblinded, RCT	Germany	1	201	AP monophasic or biphasic	5	50, 100, 200, 300, 360	Cardioversion success^a^	Persistent AF with an indication for external cardioversion, documented AF prior to the procedure, no evidence of LA thrombus, ≥ 3 weeks of anti‐coagulation

Abbreviations: AA, antero‐apical; AF, atrial fibrillation; AP, antero‐posterior; BMI, body mass index; ECG, electrocardiogram; LA, left atrial; *N*, number; RCT, randomized controlled trial.

^a^ Successful termination of AF and presence of sinus rhythm.

**TABLE 2:** Baseline characteristics of the participants.

Study ID	Arms	*N*.	Age, Years	Male, N. (%)	BMI, kg/m^2^	AF duration, months	Past history, *N*. (%)		Echocardiography		Antiarrhythmics,^a^ *N*. (%)	
							Hypertension	Diabetes	Left atrial area, cm^2^	LVEF, %	Class I (Flecainide)	Class III^b^
Ferreira et al. (2024)	Active compression	158	66.1 ± 10.5	118 (75)	32.6 ± 7	11 ± 15.7	NM	NM	NM	46.6 ± 13.8	NM	NM
	No compression	153	66.5 ± 11.3	116 (76)	31.1 ± 6.1	14 ± 22.5	NM	NM	NM	48.8 ± 13.1	NM	NM
Squara et al. (2021)	Active compression	50	70.8 ± 10.3	25 (50)	28 ± 4.9	5.8 ± 10.3	28 (56)	8 (16)	28.1 ± 5.1	45.9 ± 14	3 (6)	17 (34)
	No compression	50	69.6 ± 10.2	31 (62)	28.9 ± 7.7	6.1 ± 16.9	28 (56)	9 (18)	28.9 ± 4.8	49.1 ± 14.2	3 (6)	21 (42)
Voskoboinik et al. (2018)	Active compression	62	60 ± 10	44 (71)	35 ± 5	5 ± 5	31 (50)	24 (38)	28 ± 9	53 ± 10	7 (11)	29 (46)
	No compression	63	61 ± 11	47 (75)	35 ± 6	4 ± 9	26 (41)	16 (25)	28 ± 8	50 ± 12	11 (18)	33 (53)
Kirchhof et al. (2005)	Active compression	104	63 ± 1	72 (74)	27.4 ± 0.2	8.1 ± 2	NM	NM	NM	NM	19 (20)	36 (37)
	No compression	97	63 ± 1	75 (72)	27 ± 0.5	4.5 ± 0.2	NM	NM	NM	NM	11 (11)	38 (37)

*Note:* Data are presented as mean ± SD and proportions as *N*. (%).

Abbreviations: AF, atrial fibrillation; BMI, body mass index; LEVF, left ventricular ejection fraction; *N*, number; NM, not mentioned.

^a^ Chronic antiarrhythmic therapy.

^b^ Amiodarone or sotalol.

### 3.3 Risk of Bias and Certainty of Evidence

**FIGURE 2:**
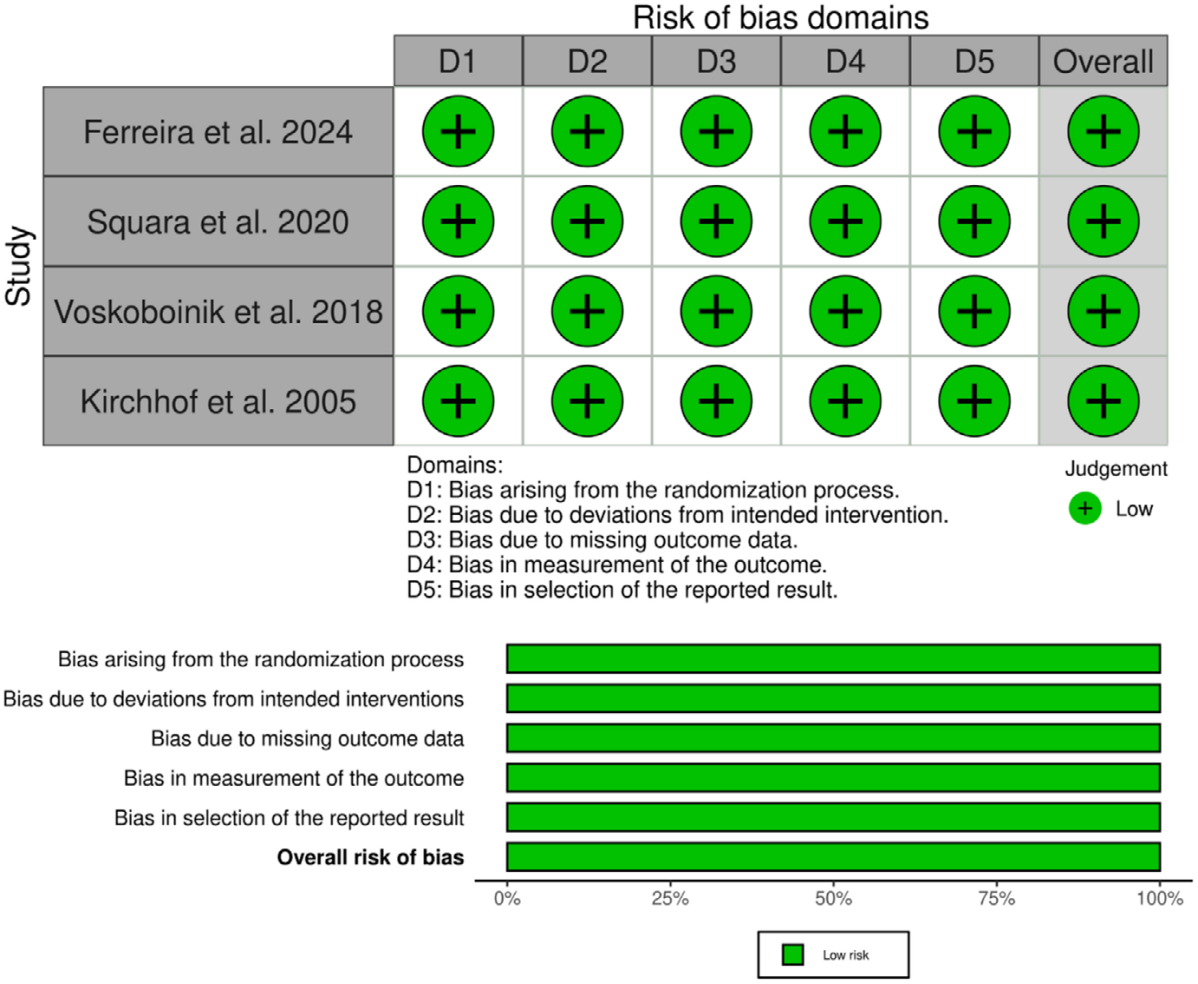
Risk of bias graph and summary of the included randomized controlled trials.

The risk of bias was assessed for each included study using the Risk of Bias 2 tool (Sterne et al. 2019). All four RCTs (Ferreira et al. 2024; Squara et al. 2021; Kirchhof et al. 2005; Voskoboinik et al. 2018) had a low overall risk of bias (Figure 2). GRADE analysis showed a low certainty of evidence with cardioversion success and successful shock energy due to limitations in imprecision. In addition, the certainty of evidence regarding first shock success, total energy delivered, number of shocks, and crossover success was very low due to limitations in imprecision and inconsistency. The full details can be found in Table A.3 in Appendix S1.

### 3.4 Primary Outcome: Cardioversion Success

**FIGURE 3:**
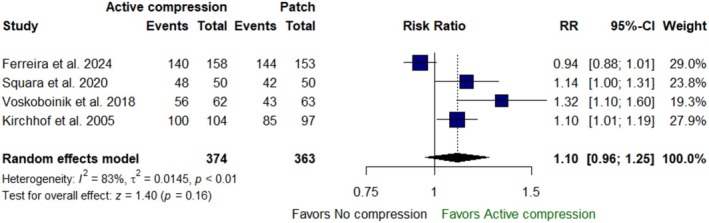
Forest plot of cardioversion success; CI, confidence interval; RR, risk ratio.

Active compression during DCCV was not associated with any significant difference in cardioversion success when compared to the no‐compression approach [RR: 1.10 with 95% CI (0.96, 1.25), *p* = 0.16] (Figure 3). Our analysis revealed a significant heterogeneity (*I* ^2^ = 83%, *p* < 0.01). Sensitivity analysis showed that the heterogeneity was best resolved after omitting the study by Ferreira et al. (2024) (*I* ^2^ = 38%) (Figure A.1 in Appendix S1).

### 3.5 Secondary Outcomes

**FIGURE 4:**
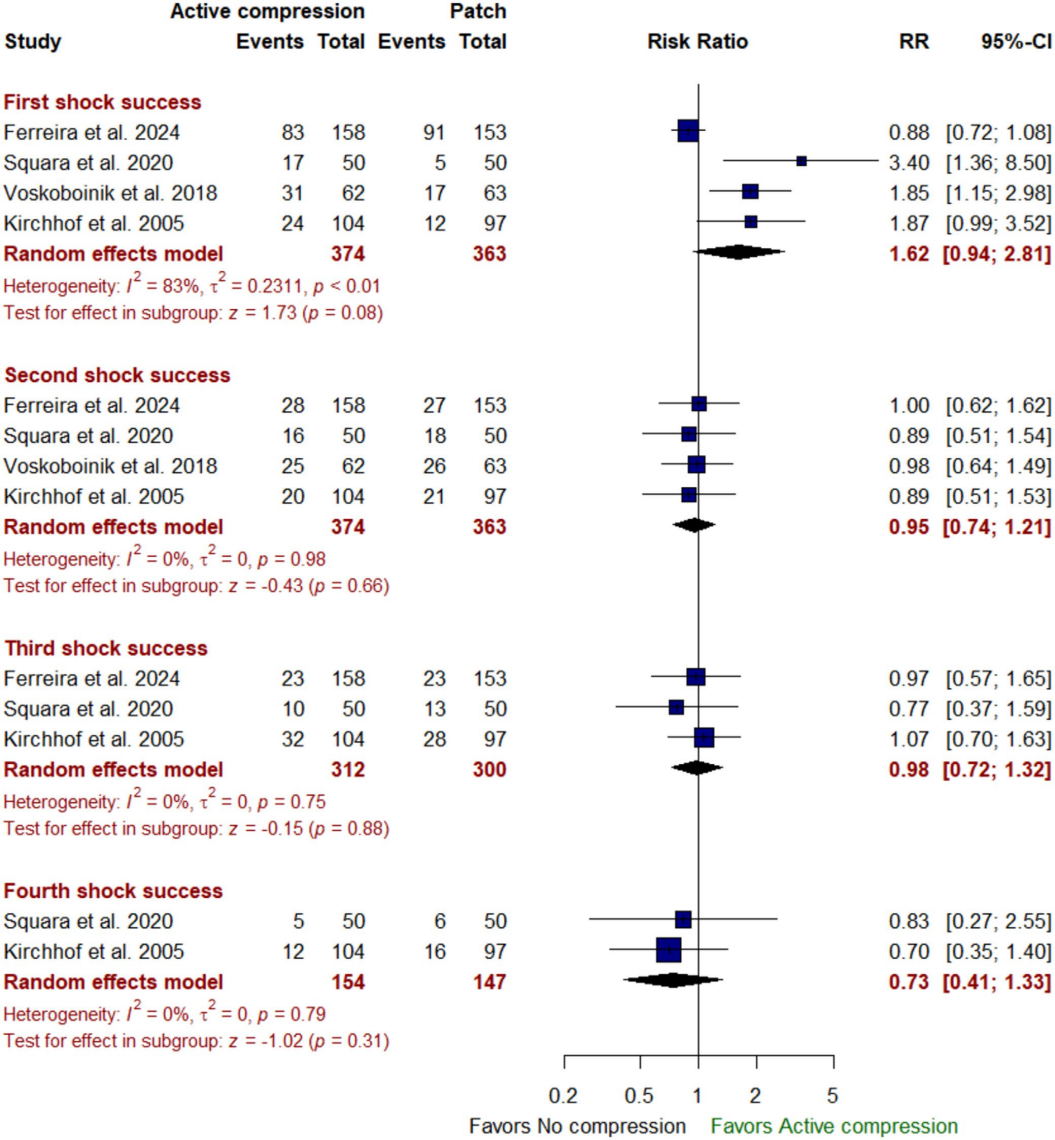
Forest plot of first, second, third, and fourth shock success; CI, confidence interval; RR, risk ratio.

When compared to the no‐compression approach, active compression during DCCV was not associated with any significant difference in first shock success [RR: 1.62 with 95% CI (0.94, 2.81), *p* = 0.08], second shock success [RR: 0.95 with 95% CI (0.74, 1.21), *p* = 0.66], third shock success [RR: 0.98 with 95% CI (0.72, 1.32), *p* = 0.88], or fourth shock success [RR: 0.73 with 95% CI (0.41, 1.33), *p* = 0.31] (Figure 4). Moreover, there was also no significant difference between the two groups regarding the number of shocks [MD: −0.32 with 95% CI (−1.01, 0.36), *p* = 0.36] (Figure A.2 in Appendix S1) or crossover success [RR: 0.76 with 95% CI (0.33, 1.77), *p* = 0.52] (Figure A.3 in Appendix S1).

**FIGURE 5:**
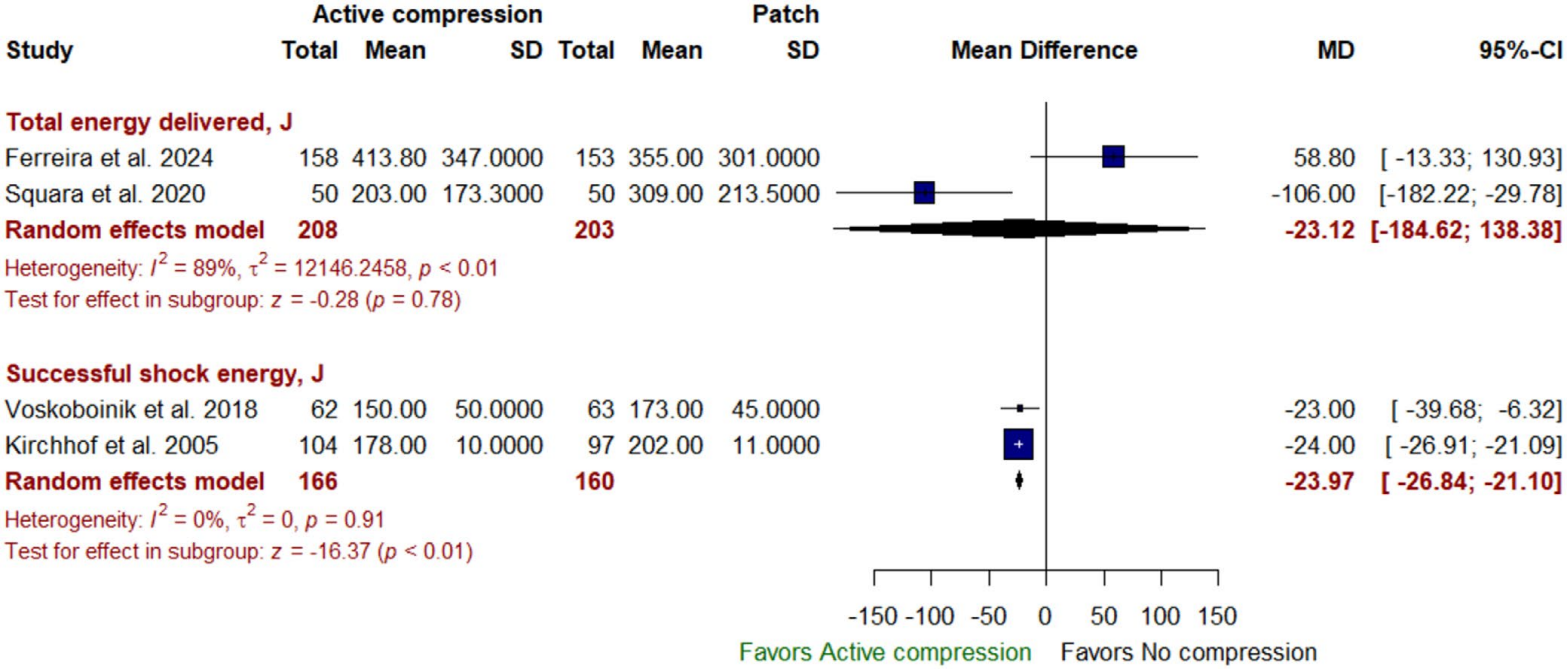
Forest plot of total energy delivered, J, and successful shock energy, J, CI, confidence interval; J, Joules; MD, mean difference.

Regarding energy used, active compression was not associated with any significant difference in total energy delivered [MD: −23.12 J with 95% CI (−184.62, 138.38), *p* = 0.78]. However, active compression was associated with a reduced successful shock energy [MD: −23.97 J with 95% CI (−26.84, −21.10), *p* < 0.01] (Figure 5).

Pooled data were homogeneous in second shock success (*I* ^2^ = 0%, *p* = 0.98), third shock success (*I* ^2^ = 0%, *p* = 0.75), fourth shock success (*I* ^2^ = 0%, *p* = 0.79), crossover success (*I* ^2^ = 0%, *p* = 0.71), and successful shock energy (*I* ^2^ = 0%, *p* = 0.91). However, pooled study data revealed substantial heterogeneity in first shock success (*I* ^2^ = 83%, *p* < 0.01) and number of shocks (*I* ^2^ = 86%, *p* < 0.01). Sensitivity analysis of first shock success revealed that the heterogeneity was best resolved after omitting the study by Ferreira et al. (*I* ^2^ = 0%) (Figure A.4 in Appendix S1). Finally, sensitivity analysis of the number of shocks was not applicable.

### 3.6 Meta‐Regression Analysis Based on BMI

There was no significant association between BMI and cardioversion success (*b* = 0.019, *p* = 0.92), first shock success (*b* = −0.051, *p* = 0.54), or second shock success (*b* = 0.011, *p* = 0.78) (Table A.4 in Appendix S1).

## 4 Discussion

As a major risk factor for stroke and heart failure, the timely and effective restoration of normal sinus rhythm is crucial in the management of AF (Saliba and Wazni 2011). DCCV remains one of the primary interventions for converting AF back to sinus rhythm, particularly in patients where pharmacological treatments have failed or are unsuitable (Brandes et al. 2020). The success of cardioversion depends on delivering an electrical shock to the heart, and optimizing the delivery of this shock is critical to improving outcomes while minimizing risks (Goyal et al. 2024).

Traditionally, DCCV is performed without active compression, relying solely on the electrical shock to restore rhythm. However, recent interest in using active compression to enhance defibrillation efficiency has grown. The rationale behind this approach is that compression may reduce thoracic impedance, potentially facilitating better energy delivery and reducing the energy required for successful cardioversion (Squara et al. 2021; Kuppahally et al. 2009). Despite the theoretical benefits, the clinical value of active compression during DCCV remains uncertain, and its impact on outcomes like cardioversion success and patient safety has been a subject of debate.

This meta‐analysis examined the efficacy of active chest compression compared to the no‐compression approach during DCCV for AF. Our findings revealed a notable reduction in the energy required for successful shocks with active compression, indicating a potential benefit in minimizing the energy delivered during the procedure, which could hold implications for patient safety (Koneru et al. 2011).

While some studies have suggested potential mechanical advantages, our findings align with earlier work suggesting no substantial improvement in cardioversion or first shock success rates with active compression (Ferreira et al. 2024). The absence of significant differences in shock success across successive attempts further reinforces the notion that the mechanical effects of compression do not translate into clinically meaningful improvements in restoring sinus rhythm. Nonetheless, our analysis showed a significant reduction in the energy required for successful shocks, a finding that has not been consistently reported in prior studies.

One potential explanation for this reduced energy requirement may lie in the modulation of thoracic impedance during active compression, allowing for more efficient energy transfer at lower shock intensities (Heyer et al. 2022; Niles et al. 2010). Although this did not translate into improved shock success, it presents an essential consideration for patient safety, particularly in cases where repeated high‐energy shocks are associated with adverse effects. Excessive energy delivery during DCCV is known to increase the risk of complications such as myocardial injury, skin burns, and hemodynamic instability (Roman et al. 2024; Nguyen et al. 2023; Bonfanti et al. 2019; Gallagher et al. 2008). As such, achieving successful cardioversion with lower energy levels could mitigate these risks, particularly in patients with compromised cardiovascular health or those undergoing repeated cardioversion attempts.

Our results highlight the importance of considering patient safety beyond immediate cardioversion success. While the clinical significance of reducing energy requirements may not be immediately apparent regarding cardioversion outcomes, it could influence long‐term patient recovery and complication rates. In clinical practice, this energy reduction could justify the use of active compression, particularly in vulnerable populations such as the elderly or those with structural heart disease, where minimizing myocardial stress is paramount (Roman et al. 2024; Franklin et al. 2022).

Heterogeneity in the pooled data, particularly regarding first shock success and overall cardioversion success, highlights significant variations across the included studies. The resolution of heterogeneity in these outcomes following the exclusion of the Ferreira et al. study raises important questions about the influence of study design, DCCV protocols, and patient characteristics on the results. Differences in shock delivery protocols may further contribute to this variability. In the Ferreira et al. study, active compression was applied during each shock using plastic gloves and a folded towel placed on the anterior chest. This approach contrasts with the approaches used in the rest of the included studies (Squara et al. 2021; Kirchhof et al. 2005; Voskoboinik et al. 2018), where compression was applied using paddles. These methodological discrepancies emphasize the need for future studies to adopt standardized chest compression and energy delivery techniques, thereby reducing variability and improving consistency in outcomes across trials.

In our analysis, BMI did not demonstrate a statistically significant association with cardioversion success, first shock success, or second shock success. The slope estimate for cardioversion success indicates a negligible effect with a wide confidence interval crossing zero. Similarly, first shock success and second shock success suggest no meaningful relationship. Our findings indicate that BMI is unlikely to play a major role in determining the success of defibrillation. These results are consistent with previous studies suggesting that body habitus alone may not significantly influence the efficacy of DCCV (Lacoste et al. 2023). However, further research with larger sample sizes and stratified analyses is necessary to confirm these findings and explore potential interactions with other clinical factors.

Another important finding was the lack of significant differences in secondary outcomes, including the required shocks and the crossover success rates between the two techniques. Despite the potential for active compression to improve energy efficiency, it did not appear to influence the total number of shocks administered, nor did it significantly alter crossover success rates. This finding suggests that while active compression might enhance the energy dynamics of DCCV, it does not provide an advantage in terms of shock efficiency.

Left atrial size is a well‐established determinant of electrical cardioversion success, as atrial enlargement has been associated with structural remodeling, increased arrhythmogenic potential, and a reduced likelihood of maintaining sinus rhythm (Abhayaratna et al. 2006). Notably, two of the referenced studies did not report left atrial size, which may have influenced the overall findings of this analysis. The absence of this critical parameter limits the ability to ascertain whether variations in cardioversion success rates across studies were attributable to differences in atrial dimensions. Similarly, left ventricular ejection fraction (LVEF) is a key prognostic indicator in cardioversion outcomes, particularly in patients with concomitant heart failure (Breathett et al. 2016). One of the included studies did not report LVEF, potentially restricting the capacity to evaluate its role in cardioversion efficacy. Given the well‐documented impact of both left atrial size and LVEF on cardioversion outcomes, the omission of these variables in some studies may have introduced residual confounding, thereby influencing the overall interpretation of our results.

## 5 Limitations

Several limitations of this meta‐analysis should be acknowledged. First, heterogeneity in the primary outcomes, even after sensitivity adjustments, suggests that variability in patient populations, DCCV protocols, and operator experience may have influenced the results. Additionally, while we identified energy reduction as a significant benefit of active compression, the clinical implications of this finding warrant further exploration, particularly concerning long‐term patient outcomes. Moreover, our sensitivity analysis demonstrated that the observed heterogeneity could be partially attributed to variations in study methodology, reinforcing the need for standardized protocols in future investigations. Finally, the small number of available studies may limit the generalizability of our findings. Also, as the number of included RCTs was (< 10), the results of the meta‐regression analysis are not reliable, warranting further investigation of the association between BMI and the effect of active compression on DCCV success.

## 6 Implications for Future Research

Future research should address the gaps in current knowledge by investigating the long‐term efficacy and safety of active compression during DCCV. RCTs incorporating standardized energy protocols and compression techniques are necessary to clarify the role of active compression, particularly in high‐risk populations. Furthermore, studies should explore the potential benefits of reduced energy delivery regarding myocardial protection and overall patient morbidity and mortality. Understanding whether the energy reduction observed in our analysis translates into measurable clinical improvements will be critical for informing future clinical guidelines and practice. Future studies should aim for standardized reporting of echocardiographic parameters, including left atrial size and LVEF, to enable more precise adjustments for these critical factors. A more comprehensive assessment of these variables may provide further insights into their role in predicting cardioversion success and help refine patient selection criteria for optimal outcomes.

## 7 Conclusion

This meta‐analysis demonstrates that active chest compression does not significantly improve cardioversion or shock success rates during DCCV for atrial fibrillation. However, the reduction in energy required for successful shocks presents a compelling argument for the potential safety benefits of this technique, particularly in reducing the risk of energy‐related complications. While immediate cardioversion success remains unchanged, the long‐term clinical implications of energy reduction merit further investigation, and future studies should focus on elucidating the full range of benefits and risks associated with active compression during DCCV.

## Author Contributions

**Hosam I. Taha:** investigation, writing – original draft, and project administration. **Abubakar Nazir:** writing – original draft. **Ahmed A. Ibrahim:** formal analysis and writing – original draft. **Mohamed S. Elgendy:** investigation and writing – original draft. **Abdalhakim Shubietah:** writing – original draft. **Hazem Reyad Mansour:** investigation and writing – original draft. **Sherif Sary:** investigation and writing – original draft. **Moataz Maged:** investigation and writing – review and editing. **Mustafa Turkmani:** writing – review and editing and supervision. **Mohamed Abuelazm:** conceptualization, writing – review and editing, and supervision. All authors have read and agreed to the final version of the manuscript.

## Ethics Statement

The authors have nothing to report.

## Consent

The authors have nothing to report.

## Conflicts of Interest

The authors declare no conflicts of interest.

## Supporting information


**Table A.1** Search strategy.
**Table A.2** List of excluded studies during the full‐text screening process.
**Table A.3** Summary of the GRADE system.
**Table A.4** Meta‐regression analysis based on BMI.
**Figure A.1** Leave‐one‐out sensitivity analysis of cardioversion success.
**Figure A.2** Forest plot of number of shocks; MD, mean difference; CI, confidence interval.
**Figure A.3** Forest plot of crossover success; RR, risk ratio; CI, confidence interval.
**Figure A.4** Leave‐one‐out sensitivity analysis of first shock success.

## Data Availability

All data are available within the manuscript and can be obtained from the corresponding author upon a reasonable request.
