# Supplementary material for: Active Compression During External Cardioversion of Atrial Fibrillation: A Meta‐Analysis of Randomized Controlled Trials
Source: Ann Noninvasive Electrocardiol. 2025 Apr 1;30(3):e70074. doi: 10.1111/anec.70074 (PMC11959626; doi:10.1111/anec.70074)
Supplement: Supplementary file 1 — Table A.1 Search strategy. Table A.2 List of excluded studies during the full‐text screening process. Table A.3 Summary of the GRADE system. Table A.4 Meta‐regression analysis based on BMI. Figure A.1 Leave‐one‐out sensitivity analysis of cardioversion success. Figure A.2 Forest plot of number of shocks; MD, mean difference; CI, confidence interval. Figure A.3 Forest plot of crossover success; RR, risk ratio; CI, confidence interval. Figure A.4 Leave‐one‐out sensitivity analysis of first shock success. [file ANEC-30-e70074-s001.docx]

**Supplementary Tables and Figures Legends.**

**Table A.1** Search strategy.

**Table A.2** List of excluded studies during the full-text screening process.

**Table A.3** Summary of GRADE system.

**Table A.4** Meta-regression analysis based on BMI.

**Figure A.1** Leave-one-out sensitivity analysis of cardioversion success.

**Figure A.2** Forest plot of number of shocks; MD: mean difference; CI: confidence interval.

**Figure A.3** Forest plot of crossover success; RR: risk ratio; CI: confidence interval.

**Figure A.4** Leave-one-out sensitivity analysis of first shock success.

**Table A.1** Search strategy

| **Database** | **Restrictions** | **Access date** | **Search strategy** | **Number of results** |
| --- | --- | --- | --- | --- |
| **PubMed** | Title/abstract | 24/9/2024 | (manual OR "manual pressure" OR compression OR "chest compression" OR pressure OR "chest pressure" OR patch* OR paddle*) AND (cardioversion OR defibrillation OR "DC shock" OR "electric shock" OR "external cardioversion") AND (AF OR "atrial fibrillation" OR "AFib") | 358 |
| **Cochrane** | Title Abstract Keyword | 24/9/2024 |  | 191 |
| **Scopus** | Title or Abstract | 24/9/2024 |  | 418 |
| **Web of Science** | Topic | 24/9/2024 |  | 512 |
| **Embase** | Title | 24/9/2024 | (manual OR (manual pressure) OR compression OR (chest compression) OR pressure OR (chest pressure) OR patch* OR paddle*) AND (cardioversion OR defibrillation OR (DC shock) OR (electric shock) OR (external cardioversion)) AND (AF OR (atrial fibrillation) OR (AFib)) | 923 |
| **Total** | | | | 2402 |

**Table A.2** List of excluded studies during the full-text screening process.

| Study ID | Title | Reason of exclusion |
| --- | --- | --- |
| Dodd et al 2004 | External defibrillation in the left lateral position—a comparison of manual paddles with self-adhesive pads | Does not meet the criteria – wrong PICO |
| Ramirez et al 2016 | Effect of Applying Force to Self-Adhesive Electrodes on Transthoracic Impedance: Implications for Electrical Cardioversion | Does not meet the criteria – wrong PICO |
| Lakananurak et al 2022 | Pad type and manual pressure augmentation with maximum output shocks in atrial fibrillation cardioversion: a randomized controlled trial (PRESS-AF study) | Does not meet the criteria – conference abstract |
| Ferreira et al 2021 | Investigating the efficacy of chest pressure for direct current cardioversion in atrial fibrillation: a randomised control trial protocol (Pressure-AF) | Does not meet the criteria – protocol |
| Forgione et al 2000 | Comparison with different site of adhesive pad electrodes versus hand-held paddle electrodes for elective cardioversion of atrial fibrillation | Does not meet the criteria – conference abstract |
| Kerber et al 1981 | Transthoracic resistance in human defibrillation. Influence of body weight, chest size, serial shocks, paddle size and paddle contact pressure. | Does not meet the criteria – wrong study design |
| Cohen et al 1997 | Active Compression Cardioversion for Refractory Atrial Fibrillation | Does not meet the criteria – wrong study design |

**Table A.3** Summary of GRADE system.

| **Certainty assessment** | | | | | | | **№ of patients** | | **Effect** | | **Certainty** | **Importance** |
| --- | --- | --- | --- | --- | --- | --- | --- | --- | --- | --- | --- | --- |
| **№ of studies** | **Study design** | **Risk of bias** | **Inconsistency** | **Indirectness** | **Imprecision** | **Other considerations** | **active compression** | **self-adhesive patch** | **Relative (95% CI)** | **Absolute (95% CI)** |  |  |
| **Cardioversion success** | | | | | | | | | | | | |
| 4 | randomised trials | not serious | serious^a^ | not serious | serious^b^ | none | 344/374 (92.0%) | 314/363 (86.5%) | **RR 1.10** (0.96 to 1.25) | **87 more per 1,000** (from 35 fewer to 216 more) | ⨁⨁◯◯ Low^a,b^ | CRITICAL |
| **Total energy delivered, J** | | | | | | | | | | | | |
| 2 | randomised trials | not serious | very serious^a^ | not serious | very serious^b,c^ | none | 208 | 203 | - | MD **23.12 lower** (184.62 lower to 138.38 higher) | ⨁◯◯◯ Very low^a,b,c^ | CRITICAL |
| **Successful shock energy, J** | | | | | | | | | | | | |
| 2 | randomised trials | not serious | not serious^d^ | not serious | very serious^e^ | none | 166 | 160 | - | MD **23.97 lower** (26.84 lower to 21.1 lower) | ⨁⨁◯◯ Low^d,e^ | CRITICAL |
| **Number of shocks** | | | | | | | | | | | | |
| 2 | randomised trials | not serious | very serious^a^ | not serious | very serious^c^ | none | 208 | 203 | - | MD **0.32 lower** (1.01 lower to 0.36 higher) | ⨁◯◯◯ Very low^a,c^ | CRITICAL |
| **First shock success** | | | | | | | | | | | | |
| 4 | randomised trials | not serious | serious^a^ | not serious | very serious^c^ | none | 155/374 (41.4%) | 125/363 (34.4%) | **RR 1.62** (0.94 to 2.81) | **213 more per 1,000** (from 21 fewer to 623 more) | ⨁◯◯◯ Very low^a,c^ | CRITICAL |
| **Second shock success** | | | | | | | | | | | | |
| 4 | randomised trials | not serious | not serious^d^ | not serious | serious^b^ | none | 89/374 (23.8%) | 92/363 (25.3%) | **RR 0.95** (0.74 to 1.21) | **13 fewer per 1,000** (from 66 fewer to 53 more) | ⨁⨁⨁◯ Moderate^b,d^ | CRITICAL |
| **Third shock success** | | | | | | | | | | | | |
| 3 | randomised trials | not serious | not serious^d^ | not serious | serious^b^ | none | 65/312 (20.8%) | 64/300 (21.3%) | **RR 0.98** (0.72 to 1.32) | **4 fewer per 1,000** (from 60 fewer to 68 more) | ⨁⨁⨁◯ Moderate^b,d^ | IMPORTANT |
| **Fourth shock success** | | | | | | | | | | | | |
| 2 | randomised trials | not serious | not serious^d^ | not serious | extremely serious^b,e,f^ | none | 17/154 (11.0%) | 22/147 (15.0%) | **RR 0.73** (0.41 to 1.33) | **40 fewer per 1,000** (from 88 fewer to 49 more) | ⨁◯◯◯ Very low^b,d,e,f^ | IMPORTANT |
| **Cross-over success** | | | | | | | | | | | | |
| 2 | randomised trials | not serious | not serious^d^ | not serious | extremely serious^f^ | none | 3/8 (37.5%) | 16/28 (57.1%) | **RR 0.76** (0.33 to 1.77) | **137 fewer per 1,000** (from 383 fewer to 440 more) | ⨁◯◯◯ Very low^d,f^ | NOT IMPORTANT |

**CI:** confidence interval; **MD:** mean difference; **RR:** risk ratio

*Explanations*

a. I^2 > 50%; shows significant heterogeneity

b. Crossing of no effect line, not excluding the risk of appreciable benefit/harm

c. Wide CI and cross no effect line with acceptable sample size.

d. I^2 < 50%; shows no significant heterogeneity

e. Small sample size (less than 450).

f. Wide CI, not excluding the risk of appreciable benefit/harm

**Table A.4** Meta-regression analysis based on BMI.

| Variables | Slope point estimate (lower limit to upper limit) | P-value | Slope point estimate (lower limit to upper limit) | P-value | Slope point estimate (lower limit to upper limit) | P-value |
| --- | --- | --- | --- | --- | --- | --- |
|  | **Defibrillation success** | | **First shock success** | | **Second shock success** | |
| **BMI** | 0.019 (-0.039, 0.043) | 0.92 | -0.051 (-0.221, 0.117) | 0.54 | 0.011 (-0.071, 0.095) | 0.78 |

**
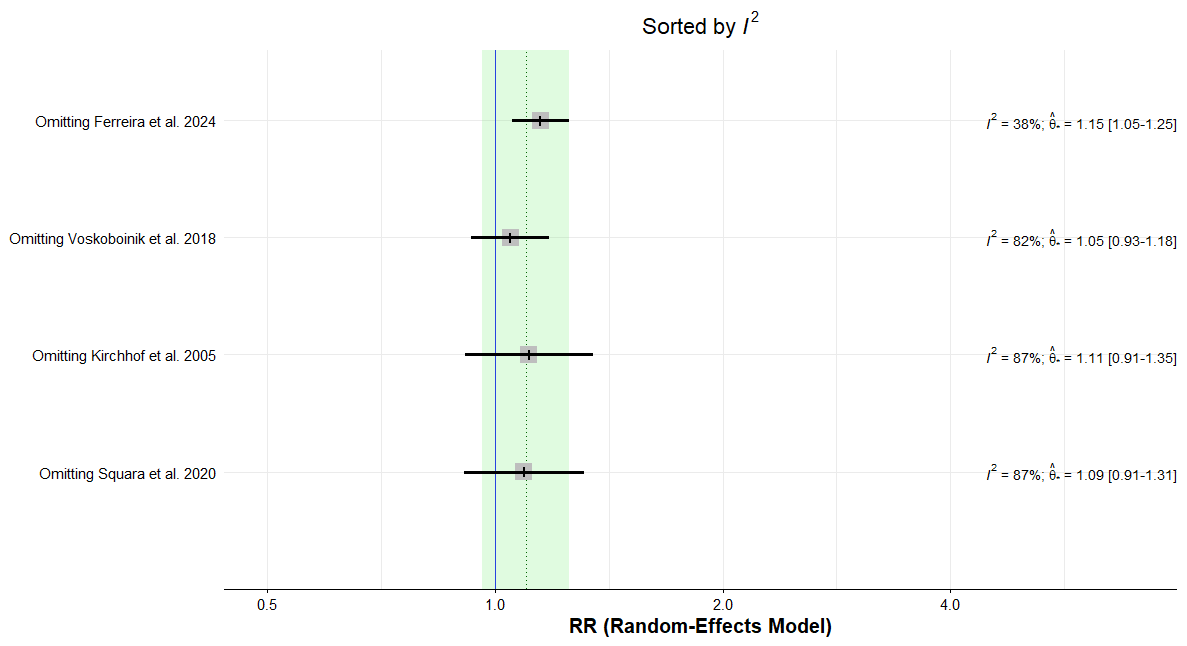
**

**Fig. A.1** Leave-one-out sensitivity analysis of cardioversion success.

*
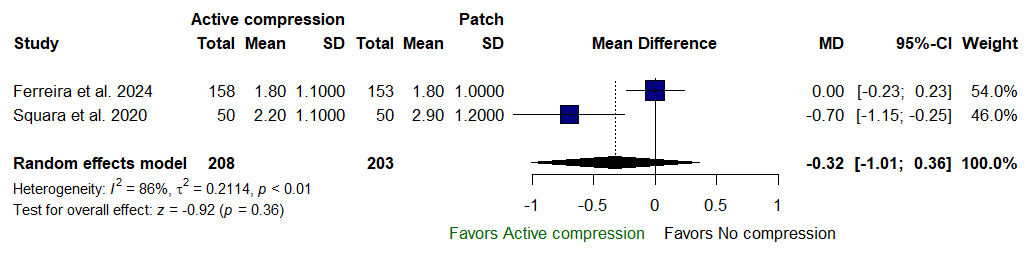
*

**Fig. A.2** Forest plot of number of shocks; MD: mean difference; CI: confidence interval.


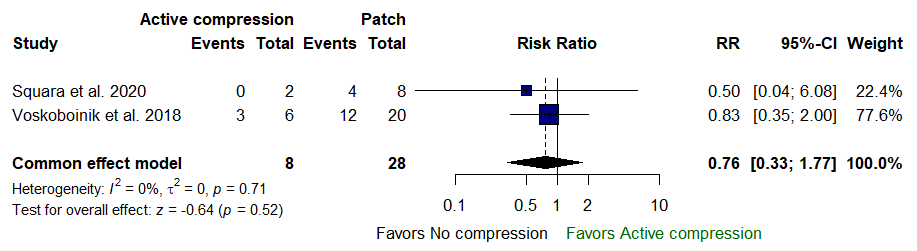


**Fig. A.3** Forest plot of crossover success; RR: risk ratio; CI: confidence interval.

**
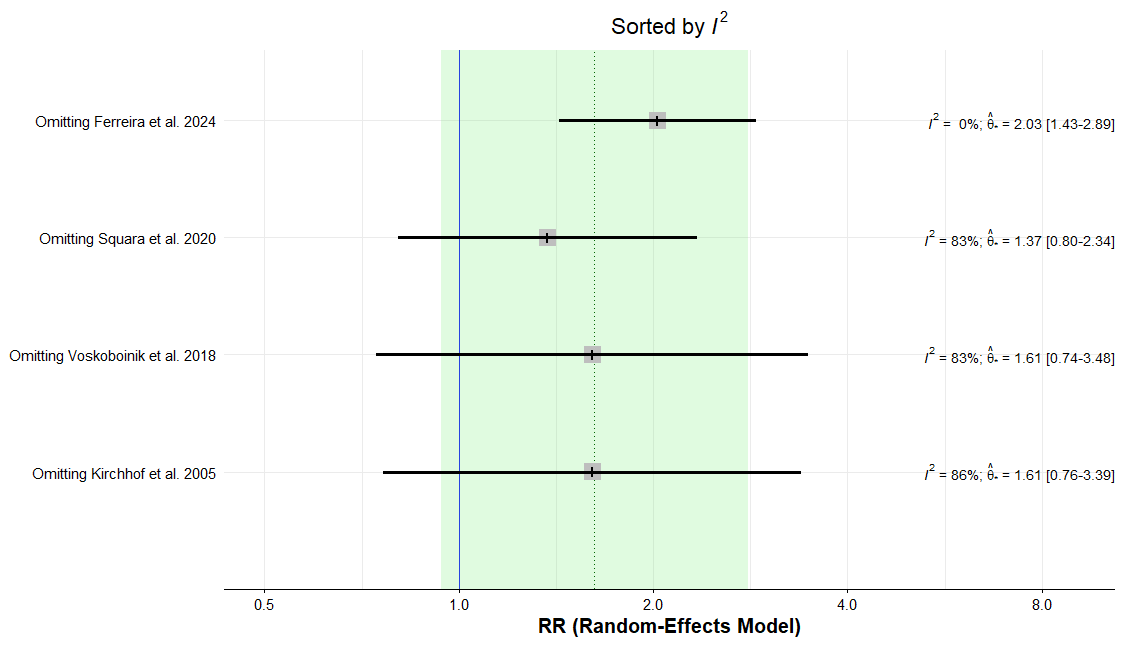
**

**Fig. A.4** Leave-one-out sensitivity analysis of first shock success.
